# Discrimination of hospital isolates of *Acinetobacter baumannii* using repeated sequences and whole genome alignment differential analysis

**DOI:** 10.1007/s13353-021-00640-5

**Published:** 2021-06-08

**Authors:** Roman Kotłowski, Alicja Nowak-Zaleska, Grzegorz Węgrzyn

**Affiliations:** 1 Department of Molecular Biotechnology and Microbiology, Faculty of Chemistry, Gdansk University of Technology, Gabriela Narutowicza 11/12 street, 80-233 Gdansk, Poland; 2 Department of Molecular Biology, Faculty of Biology, Gdansk University, Wita Stwosza 59 street, 80-308 Gdańsk, Poland

**Keywords:** *Acinetobacter baumannii*, Hospital infections, DNA polymerase III gene DNA polymerase III subunit gamma/tau, Genetic polymorphisms, Antibiotics, Assembled matrix data

## Abstract

An optimized method for bacterial strain differentiation, based on combination of Repeated Sequences and Whole Genome Alignment Differential Analysis (RS&WGADA), is presented in this report. In this analysis, 51 *Acinetobacter baumannii* multidrug-resistance strains from one hospital environment and patients from 14 hospital wards were classified on the basis of polymorphisms of repeated sequences located in CRISPR region, variation in the gene encoding the EmrA-homologue of *E. coli*, and antibiotic resistance patterns, in combination with three newly identified polymorphic regions in the genomes of *A. baumannii* clinical isolates. Differential analysis of two similarity matrices between different genotypes and resistance patterns allowed to distinguish three significant correlations (*p* < 0.05) between 172 bp DNA insertion combined with resistance to chloramphenicol and gentamycin. Interestingly, 45 and 55 bp DNA insertions within the CRISPR region were identified, and combined during analyses with resistance/susceptibility to trimethoprim/sulfamethoxazole. Moreover, 184 or 1374 bp DNA length polymorphisms in the genomic region located upstream of the GTP cyclohydrolase I gene, associated mainly with imipenem susceptibility, was identified. In addition, considerable nucleotide polymorphism of the gene encoding the gamma/tau subunit of DNA polymerase III, an enzyme crucial for bacterial DNA replication, was discovered. The differentiation analysis performed using the above described approach allowed us to monitor the distribution of *A. baumannii* isolates in different wards of the hospital in the time frame of several years, indicating that the optimized method may be useful in hospital epidemiological studies, particularly in identification of the source of primary infections.

## Introduction

The genome of each microorganism is a source of knowledge that can be applied for strain differentiation, based on bioinformatic tools and available techniques of molecular biology, suitable for epidemiological investigations. Among the species of the genus *Acinetobacter*, *A. baumannii* strains manifest the highest pathogenicity (Wong et al. 2017; Skariyachan et al. 2019). They are highly opportunistic microorganisms, responsible for hospital infections related to ability to adapt to different environmental conditions (Antunes et al. 2014). At the beginning of the twenty-first century, no complete genome sequence of *Acinetobacter* sp. was known. Barbe et al. (2004) published the first sequence of *Acinetobacter* sp. ADP1 genome, and later Smith et al. (2007) published the complete genome of *A. baumannii* ATCC 17,978. Subsequently, the first genomic sequence of the multidrug-resistant *A. baumannii* strain was published by Adams et al. (2008). Presently, complete sequences of the genomic DNA of *A. baumannii* are known for about 250 strains (http://www.ncbi.nlm.nih.gov, database retrieved on 10 December 2020). As indicated by various research teams, repeated sequences of *A. baumannii* and other microorganisms have great impact in the process of generating pathogenicity for immunocompromised hosts (Zhou et al. 2014; Shariat and Dudley 2014; Nabil et al. 2015) or adaptation skills to different environmental conditions (Zhou et al. 2014; Shariat and Dudley 2014; Karah et al. 2015).

The presence of tandem DNA repeats in genomes of *A. baumannii* was confirmed by several groups (Martín-Lozano et al. 2002; Turton et al. 2009; Irfan et al. 2011; Pourcel et al. 2011; Minandri et al. 2012; Ergin et al. 2013; Ahmed and Alp 2015; Villalón et al. 2015). Based on these sequences, different methods of differentiation of *A. baumannii* strains have been developed; however, they take into account only their diversifying power of evolutionary changes of the *Acinetobacter* genus (Touchon et al. 2014). Thus, their features responsible for drug resistance or pseudo-immunological bacterial responses, encoded in the Clustered Regularly Interspaced Short Palindromic Repeats (CRISPR) system, which evolved to protect the cells from exogenous phage and plasmid DNA invasion, are ignored in such analyses. On the other hand, as suggested by Touchon et al. (2014), the next step in the process of strains’ classification should be focused on confrontation of the genetic and phenotypic features related to pathogenicity of bacterial species. To address some of the above features, an optimized method for *A. baumannii* differential analysis is proposed in this report. It is based on combination of the previously described method based on analysis of repeated sequences (Nowak-Zaleska et al. 2008, 2016) and whole genome alignment.

## Materials and methods

### Bacterial strains

We used 51 *A. baumannii* isolates from diagnostic materials of the hospital environments of Antoni Jurasz University Hospital in Bydgoszcz. These isolates were derived from 11 hospital wards (Dermatology, Endocrinology, Geriatrics, General and Endocrine Surgery, General and Vascular Surgery, Intensive Care Units, Neurology, Nephrology, Neurosurgery, Orthopedic, Plastic Surgery), 2 clinics (Orthopedic Outpatient Clinic, Surgical Outpatient Clinic), and Rehabilitation Department. The isolates were collected during the period of 2003–2006 (Table 1). The following strains were isolated from different diagnostic materials: 10 from bronchoalveolar lavages, 8 from bedsores, 2 from blood, 1 from cerebrospinal fluid, 2 from drains, 2 from drain swabs, 1 from needle tip, 1 from pus, 8 from respiratory secretions, 2 from tracheostomy tube swabs, 1 from tube swab, 9 from ulceration wounds, and 4 from urine. Strains were identified based on ID GN phenotypic identification system, including drug sensitivity. This identification was conducted using Kirby–Bauer method, according to CLSI instructions (for details, see Nowak-Zaleska et al. 2008, 2016).

**Table 1:** Characteristics of multidrug-resistant *Acinetobacter baumannii* clinical isolates

No	Isolates^*^	Antibiogram^a^	Genotype pattern^b^	Combined analysis cluster^c^	Source of isolates^#^
1	2005VI.70.ICU	I	1	1	Ulceration wound
2	2006III.107.NS	II	8	2	Respiratory secretion
3	2006I.96.ICU	II	8	2	BAL
4	2006I.95.ICU	II	8	2	BAL
5	2006I.93.R	II	8	2	Urine
6	2006I.92.ICU	II	8	2	BAL
7	2006II.105.E	II	7	3	Respiratory secretion
8	2006IV.108.NS	II	7	3	CSF
9	2005XI.85.ICU	II	8	2	BAL
10	2005XII.91.ICU	II	8	2	BAL
11	2005XI.88.R	II	8	2	Urine
12	2005XI.87.PS	II	8	2	Bedsores
13	2006II.98.R	II	8	2	Respiratory secretion
14	2005VI.71.R	II	10	4	Respiratory secretion
15	2006II.100.G	II	10	4	Urine
16	2006II.101.ICU	II	10	4	Blood
17	2005X.79.NS	II	10	4	Urine
18	2006II.102.ICU	II	9	5	BAL
19	2005IV.68.R	II	13	6	Drain swab
20	2003VI.43.G&ES	II	6	7	Ulceration wound
21	2003VIII.45.O	II	6	7	Drain swab
22	2003IX.48.N	II	6	7	Tracheostomy tube swab
23	2004XI.61.O	II	15	8	Ulceration wound
24	2004X.59.OC	III	4	9	Bedsores
25	2006I.94.NS	IV	8	10	Respiratory secretion
26	2006II.104.NS	IV	7	11	Respiratory secretion
27	2004VIII.55.OC	V	2	12	Bedsores
28	2003XI.50.O	V	6	13	Bedsores
29	2005I.65.O	V	4	14	Drain
30	2003IX.47.ICU	VI	6	15	BAL
31	2005VIII.72.G&ES	VI	15	16	Ulceration wound
32	2003VIII.44.ICU	VII	6	17	Ulceration wound
33	2003IX.46.G&ES	VII	6	17	Ulceration wound
34	2003III.42.ICU	VII	12	18	Tracheostomy tube swab
35	2003IX.49.D	VIII	14	19	Ulceration wound
36	2005IV.67.ICU	IX	15	20	Ulceration wound
37	2004IV.52.E	X	15	21	Bedsores
38	2006II.103.ICU	X	15	21	BAL
39	2004X.58.R	X	15	21	Tube swab
40	2005III.66.O	XI	4	22	Drain
41	2004X.56.NS	XII	11	23	Blood
42	2004X.57.NS	XII	15	24	Bedsores
43	2004XI.63.R	XIII	3	25	Pus
44	2004VIII.54.ICU	XIII	5	26	BAL
45	2004XI.62.G	XIII	4	27	Bedsores
46	2005 V.69.SC	XIII	15	28	Ulceration wound
47	2004VI.53.N	XIV	16	29	Bedsores
48	2006II.106.NS	XV	8	30	Respiratory secretion
49	2005XII.90.Nef	XV	8	30	Respiratory secretion
50	2005IX.76.ICU	XV	10	31	BAL
51	2005IX.78.G&VS	XV	10	31	Needle tip
HGDI index		0.8	0.8816	0.9718	

^a^For details of particular antibiogram patterns, see Table 5

^b^For details of particular genotype patterns, see Table 4

^c^Numbers arisen from combination of antibiogram and genotype patterns

^*^Abbreviations for isolates (the last letter(s) in the name): D—Dermatology, E—Endocrinology, G—Geriatrics, G&ES—General and Endocrine Surgery, G&VS—General and Vascular Surgery, ICU—Intensive Care Unit, N—Neurology, Nef—Nephrology, NS—Neurosurgery, O—Orthopedic, OC—Orthopedic Outpatient Clinic, PS—Plastic Surgery, R—Rehabilitation, SC—Surgical Outpatient Clinic

^#^Abbreviations for source of isolates: BAL—bronchoalveolar lavage; CSF—cerebrospinal fluid

### Locus identification with repeated sequences

The isolates of *A. baumannii* were differentiated on the basis of previously published polymorphisms of repeated sequences located in the CRISPR region (Touchon et al. 2014), variation in the gene encoding the EmrA homologue of *E. coli* (Nowak-Zaleska et al. 2016), and three newly identified (in this study) polymorphic regions (Tables 2 and 3).

**Table 2:** The sizes of PCR products for designed pairs of primers calculated for selected *Acinetobacter baumannii* genomes

Genome NCBI accession numbers* of *Acinetobacter baumannii* strains	PCR product length (bp)		
	Genomic region 1	Genomic region 2	Genomic region 3
	Primer pairs: Aci7 and Aci8	Primer pairs: Aci13 and Aci14	Primer pairs: Aci17 and Aci18
CP001172.2 *Acinetobacter baumannii* AB307-0294	204	184	404
NC_011586.2 *Acinetobacter baumannii* AB0057	162	184	405
CP002522.2 *Acinetobacter baumannii* TCDC-AB0715	180	236	508
NC_010611.1 *Acinetobacter baumannii* ACICU	144	1274	508
CP001937.2 *Acinetobacter baumannii* MDR-ZJ06	222	1374	500
CP003500.1 *Acinetobacter baumannii* MDR-TJ	222	1374	508
CP003847.1 *Acinetobacter baumannii* BJAB0715	156	186	406
NZ_CP018664.1 *Acinetobacter baumannii* ATCC 17,978	210	185	306
NC_010410.1 *Acinetobacter baumannii* AYE	234	1373	405

^*^NCBI—National Center for Biotechnology Information

**Table 3:** Identification of proteins within amplified genomic regions of *Acinetobacter baumannii* MDR-TJ strain

No. of genomic regions	Location of PCR product	Location of PCR product within *Acinetobacter baumannii* MDR-TJ genome, GenBank: CP003500.1			
1	Aci7 and Aci8 1,558,399–1,558,566 bp	ACI7 5′GTGCTGTTCAGCCTGTTGAAGTTATTAG ACI8 5′CAACTGCTGACTCAAGTCCAATCAACTC			
		Locus_tag = "ABTJ_01493" Product = "DNA polymerase III, subunit gamma/tau" Protein_id = "AFI95102.1" 1,557,159..1559279 bp			
2	Aci13 and Aci14 1,197,192–1,198,491 bp	ACI13 5′GAGGTACTAAAAATAAAAAGCGGGGATAAAAGTAGACAAG ACI14 5′GTTGGGCTTTTTTTATAGCTGAACGCGATAAACTTC			
		Locus_tag = "ABTJ_01149" "Signal predicted by SignalP 3.0 HMM; IMG reference gene:2510836153_SP" Product = "hypothetical protein" Protein_id = "AFI94769.1" 1,196,033..1197184 bp	Locus_tag = "ABTJ_01151" Product = "hypothetical protein" Protein_id = "AFI94772.1" 1,197,921..1198355 bp		Locus_tag = "ABTJ_01152" Product = "GTP cyclohydrolase I" 1,198,535..1199089 bp
3	Aci17 and Aci18 1,707,347–1,707,791 bp	ACI17 5′CAGTTTAAACAGGTGTCAAATCGTAAACAAATATTGATG ACI18 5′GGCAGAAACTAGCCACGATGCAAGCA			
		Locus_tag = "ABTJ_01661" Product = "Protein of unknown function (DUF2750)" 1,706,849..1707274 bp		Locus_tag = "ABTJ_01662" Product = "hypothetical protein" Protein_id = "AFI95267.1" 1,707,761..1707994 bp	

### DNA-technology methods

The genetic material from the isolates was obtained using Genomic Mini Set, purchased from A&A Biotechnology (Gdynia, Poland), following the manufacturer’s instruction. For the DR-PCR/RFLP genotyping method, sequences of primers, the PCR reaction conditions, and enzymatic digestion of PCR products were previously described (Nowak-Zaleska et al. 2008). Briefly, the amplification reactions were conducted according to the following time–temperature profile: 94 °C for 2 min, during the initial denaturation step, 35 cycles consisting of the DNA denaturation at 94 °C for 1 min, hybridization at 68 °C for 1 min, and extension at 72 °C for 2 min. The amplification products were subjected to the restriction fragment length polymorphism (RFLP) analysis using *Hae*III and *Ssi*I restriction enzymes. Separation of restriction fragments was performed electrophoretically, in 12% polyacrylamide gels, and results were documented using Versa Doc Imaging System, ver. 1000. The homologous region of the *emrA* resistance-related gene, containing 6-nt repeats, was analyzed as described previously (Nowak-Zaleska et al. 2016). Identification of three newly discovered polymorphic regions was possible after multiple alignment of nine *A. baumannii* genomes (see Table 2), using the MAFFT 7.271 software (Katoh et al. 2002). Subsequently, three pairs of primers, shown in Table 3, were used in the PCR analysis. The PCR reactions were conducted in 25 μl reaction mixtures, using the Eppendorf AG 22,331 thermal cycler. The PCR mixtures were as follows: 1.5 U of RUN DNA polymerase (purchased from A&A Biotechnology), PCR reaction buffer containing 10 mM KCl, 10 mM (NH_4_)_2_SO_4_, 0.1% Triton X-100, 20 mM Tris, pH 8.5, 2 mM of Mg_2_Cl, 2 mM of each deoxynucleoside triphosphates, 25 pM of suitable pairs of primers, and 50 ng/μl of template DNA. Amplified PCR products were separated using 2% agarose gel electrophoresis and standard ethidium bromide staining procedure (Sambrook et al. 1989). Images of the gels were obtained using Versa Doc Imaging System, ver. 1000.

### Statistical analysis

Statistical analysis was performed using Epi Info 7.2.3.1 software using two-tailed Fisher exact test analysis. The values “1” and “0” were representing resistant and susceptible strains for different antibiotics used in our study. Similarity matrices of different genotypes and resistance features and phylogenetic trees were constructed using package MVSP ver. 3.22.

## Results and discussion

To enhance the currently available methods of differentiation of *A. baumannii* strains, we were searching for previously unknown PCR-derived fragment length polymorphism variations in randomly identified regions of selected genomic sequences. The theoretical values of PCR fragment lengths of the newly discovered polymorphic regions for nine *A. baumannii* genomes are presented in Table 2. Among three identified polymorphic regions, only one was characterized by the highest length polymorphism. It was recognized as a gene fragment coding for DNA polymerase III subunit gamma/tau, with the Protein_id = AFI95102.1 in the MDR-TJ *A. baumannii* genome (GenBank accession no. CP003500.1) (Table 3).

In order to increase variation of analyzed *A. baumannii* isolates, two other previously described variable regions in the genomes of *A. baumannii* were included in our study (Nowak-Zaleska et al. 2008, 2016). The combined application of the three genetic aforementioned genotypic methods, DR-PCR/RFLP, different number of P-A dipeptide repeats encoded in the N-terminal part of EmrA-homologue gene, as well as three new variables, namely, Aci7 and Aci8, Aci13 and Aci14, and Aci17 and Aci18 (Table 4), combined with known information about resistance patterns for each isolate (Table 5), allowed for recognition of 31 different clusters shown in Table 1.

**Table 4:** Set of different genotypes shown as PCR length polymorphisms in nucleotide base pairs for 51 MDR *Acinetobacter baumannii* isolates

Genotypes	Three new PCR regions (length in bp)			PCR-DR/RFLP region (length in bp)															EmrA^*^—homologue gene fragment (length in bp)
	Genomic region 1 Aci7 and Aci8	Genomic region 2 Aci13 and Aci14	Genomic region 3 Aci17 and Aci18	*Hae*III pattern							*Ssi*I pattern								
				#1	#2	#3	#4	#5	#6	#7	#1	#2	#3	#4	5	#6	#7	#8	
1	156	184	600	106	0	63	60	57	54	45	0	137	109	88	76	63	43	38	138
2	234	184	405	107	83	78	64	60	59	55	0	0	111	0	74	61	43	38	126
3	204	184	405	106	82	63	60	57	54	45	0	137	109	88	76	63	43	38	126
4	210	184	405	106	82	63	60	57	54	45	0	137	109	88	76	63	43	38	126
5	234	184	405	106	82	63	60	57	54	45	0	137	109	88	76	63	43	38	126
6	222	184	405	106	82	63	60	57	54	45	0	137	109	88	76	63	43	38	126
7	234	1374	508	106	82	63	60	57	54	45	172	134	110	89	76	63	42	37	126
8	222	1374	508	106	82	63	60	57	54	45	172	134	110	89	76	63	42	37	126
9	210	1374	508	106	82	63	60	57	54	45	172	134	110	89	76	63	42	37	132
10	210	1374	508	106	82	63	60	57	54	45	172	134	110	89	76	63	42	37	126
11	180	1374	508	106	82	63	60	57	54	45	172	134	110	89	76	63	42	37	120
12	144	1374	306	109	77	71	64	58	55	0	0	137	109	88	76	63	43	38	132
13	210	1374	405	106	82	63	60	57	54	45	0	137	109	88	76	63	43	38	126
14	210	1374	405	106	82	63	60	57	54	45	0	137	109	88	76	63	43	38	132
15	156	1374	306	109	77	71	64	58	55	0	0	137	109	88	76	63	43	38	132
16	162	1374	306	109	77	71	64	58	55	0	0	137	109	88	76	63	43	38	132

^*^EmrA—an enzyme from *Escherichia coli*

^#^—restriction pattern number

**Table 5:** Set of different antibiotic resistance patterns determined for 51 MDR *Acinetobacter baumannii s*trains

Resistance pattern	Antibiotic resistance/susceptibility												
	IPM	NET	NN	CAZ	CIP	CTX	CFP	TIC	ATM	SXT	C/GM	GM/C	AN
I	R	S	R	S	R	R	R	R	R	R	R	R	R
II	S	R	R	R	R	R	R	R	R	R	R	R	R
III	S	R	S	R	R	R	R	R	R	R	R	R	R
IV	S	R	R	R	R	R	R	R	R	R	R	R	S
V	S	R	R	R	S	R	R	R	R	R	R	R	R
VI	S	S	R	R	R	R	R	R	R	R	R	R	R
VII	S	S	S	R	R	R	R	R	R	R	R	R	R
VIII	S	S	S	S	S	S	S	S	S	S	R	R	R
IX	S	S	R	R	R	R	R	I	R	R	R	R	R
X	S	S	I	R	R	R	R	I	R	R	R	R	R
XI	S	R	R	R	I	R	R	R	I	R	R	R	R
XII	S	I	R	R	R	R	R	R	R	R	R	R	R
XIII	S	R	R	R	R	R	R	I	R	R	R	R	R
XIV	S	R	I	R	R	R	R	I	R	R	R	R	R
XV	S	R	R	R	R	R	R	R	R	R	R	R	I

Meaning of symbols: R, resistance; S, susceptibility; I, intermediate phenotype

Antibiotics abbreviations: AN, amikacin; ATM, aztreonam; C, chloramphenicol; CAZ, ceftazidime; CFP, cefoperazone; CIP, ciprofloxacin; CTX, cefotaxime; GM, gentamycin; IPM, imipenem; NN, tobramycin; NET, netilmicin; SXT, trimethoprim/sulfamethoxazole; TIC, ticarcillin

Identical results for GM and C for different restriction patterns SsiI_1 and SsiI_2 are named C/GM and GM/C

Detailed analysis of bacterial isolates and diagnostic material revealed significant differences between *A. baumannii* isolates from bronchoalveolar lavage (BAL) and other clinical samples (*p* < 0.0001), as well as significant correlation between resistance pattern II and genotype 8^th^ (*p* < 0.01), presented in Table 1. In addition, significant correlation (*p* < 0.05) between the frequency of occurrence of 8^th^ *A. baumannii* genotype in the first trimester of 2006 year in comparison to other periods of isolation time was also evident. Higher Hunter–Gaston Discriminatory Index (HGDI), presented in Table 1, was determined using the method developed in this study, in comparison to previously published genotyping methods (Nowak-Zaleska et al. 2008, 2016). Furthermore, in the course of statistical data analysis, we observed that strains representing clusters 2 and 4 from combined genetic–phenotypic analysis, shown in Table 1, were isolated in two consecutive years 2005 and 2006 (*p* < 0.0001). These strains represent the 15^th^ genotype pattern, which was present in 2004 and 2005, but with different resistance patterns II, VI, IX, X, XII, and XIII (*p* = 0.01). In addition, three strains representing 21^st^ cluster with the resistance pattern X appeared in years 2004 and 2006. Moreover, strains with genotypes 6, 12, and 14 were only present in 2003 (*p* < 0.0001), in comparison to other genotypes, and what is interesting, the resistance pattern II appeared each year, while patterns V, VI, VII, and VIII appeared only between 2003 and 2005 (*p* = 0.01).

**Fig. 1:**
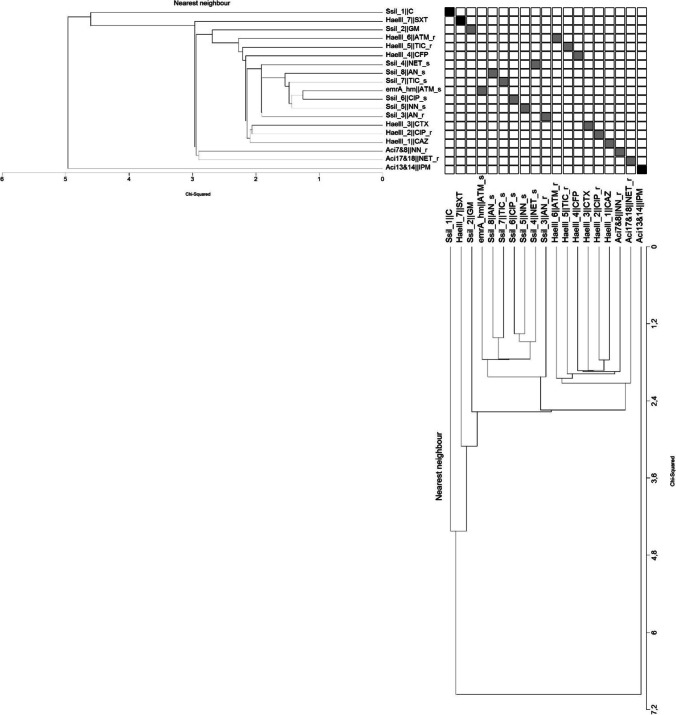
Phylogenetic trees for different pairs of genetic polymorphisms and resistance/susceptibility features. Branches order obtained based on nearest neighbor method and length–distance calculation based on *χ*^2^ method. Significant (*p* < 0.05) differences indicated in black boxes were identified based on cut-off *χ*^2^ value = 4

Combined analysis of similarity matrices, obtained using data from Tables 4 and 5, revealed that out of 19 combinations of genetic and resistance markers, only three were significantly different (*p* < 0.05) (Table 6), as indicated by *χ*^2^ value higher than 4, obtained from two phylogenetic trees presented in Fig. 1. Among significantly different mixed parameters identified, there were (1) 172 bp DNA insertion, located in the CRISPR locus, identified using the *Ssi*I enzyme for genotypes 7 to 11, in combination with resistance to chloramphenicol and gentamycin; (2) 45 and 55 bp DNA insertions in the same locus, identified using the *Hae*III enzyme, combined with trimethoprim/sulfamethoxazole resistance or susceptibility patterns; and (3) 184 or 1374 bps DNA length polymorphisms in the second genomic region (see tree new PCR region, Table 4), identified in our study for genotypes 1 to 6 and 7 to 16, in combination with imipenem resistance, characteristic for pattern I or susceptibility features, characteristic for other patterns (Table 6).

**Table 6:**
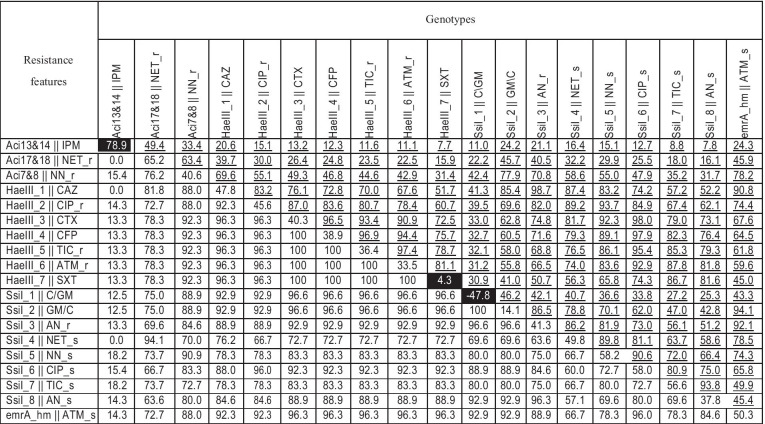
Set of two joined-similarity matrices obtained for 19 different genotypes indicated by underlined values, and for 19 different antibiotic resistance patterns. All values are from the range between 1 and 100%. Abbreviations "_s" and "_r" indicate intermediate resistance patterns considered two times as susceptible or resistant, respectively. The "0" value was replaced by "1E-06" for diagonal correlation calculation purposes. Significant (*p* < 0.05) combinations of genetic and resistance/susceptibility features are highlighted in black

The presence of *A. baumannii* genotypes over a period of 4 years in the hospital wards (Table 7), and location of determined genotypes over a 4-year period in hospital wards (Table 8), was also assessed. This analysis provides a possibility to identify the presence of specific isolates in various wards over the period of several years. We suggest that such analyses may be useful in epidemiological studies on the origin and migration of particular bacterial strains between different wards of an investigated hospital. It also gives the possibility to analyze the strains regardless of the time period in which they were collected.

**Table 7:** Presence of *A. baumannii* genotypes over a period of 4 years

Year of isolation of the strain _(number of genotypes determined)_	Ward	Genotype (number of isolates)
2006_(5)_	ICU	8 (3), 10, 9, 15
	NS	8 (3), 7 (2)
	R	8 (2)
	E	7
	G	10
2005_(6)_	ICU	1, 8 (2), 15, 10
	R	8, 10, 13
	PS	8
	NS	10
	O	4 (2)
	G&ES	15
	SC	15
	Nef	8
	G&VS	10
2004_(7)_	ICU	5
	O	15
	OC	4, 2
	E	15
	R	15, 3
	NS	11, 15
	G	4
	N	16
2003_(3)_	ICU	6 (2), 12
	G&ES	6 (2)
	O	6 (2)
	N	6
	D	14

Abbreviations for wards: D—Dermatology, E—Endocrinology, G—Geriatrics, G&ES—General and Endocrine Surgery, G&VS—General and Vascular Surgery, ICU—Intensive Care Unit, N—Neurology, Nef—Nephrology, NS—Neurosurgery, O—Orthopedic, OC—Orthopedic Outpatient Clinic, PS—Plastic Surgery, R—Rehabilitation, SC—Surgical Outpatient Clinic

**Table 8:** Location of determined genotypes over a 4-year period in hospital wards

Genotype	Year_(number of genotypes)_	Hospital ward(s)
15	2006_(1)_	ICU
	2005_(3)_	ICU, G&ES, SC
	2004_(4)_	O, E, R, NS
8	2006_(8)_	ICU, NS, R
	2005_(5)_	ICU, R, Nef
10	2006_(2)_	ICU, G
	2005_(4)_	ICU, R, NS, G&VS
4	2005_(2)_	O
	2004_(2)_	OC, G
6	2003_(7)_	ICU, G&ES, O, N
7	2006_(3)_	E
1	2005_(1)_	ICU
2	2004_(1)_	ICU
3	2004_(1)_	R
5	2004_(1)_	ICU
9	2006_(1)_	ICU
11	2004_(1)_	NS
12	2003_(1)_	ICU
13	2005_(1)_	R
14	2003_(1)_	D
16	2004_(1)_	N

Abbreviations for wards: D—Dermatology, E—Endocrinology, G—Geriatrics, G&ES—General and Endocrine Surgery, G&VS—General and Vascular Surgery, ICU—Intensive Care Unit, N—Neurology, Nef—Nephrology, NS—Neurosurgery, O—Orthopedic, OC—Orthopedic Outpatient Clinic, PS—Plastic Surgery, R—Rehabilitation, SC—Surgical Outpatient Clinic

## Conclusions

In conclusion, 16 different genotypes out of 51 MDR *A. baumannii* clinical isolates were identified in our study. Based on combined comparative analysis of genetic and resistance patterns, two significantly different patterns of DNA polymorphisms in the CRISPR coding region, resistance to chloramphenicol and gentamycin features, and resistance or susceptibility to trimethoprim/sulfamethoxazole, specific groups of isolates were identified. Out of 19 genetic markers and antibiotic resistance features, three of them were shown to be statistically significantly different using two statistical tools (Table 6, Fig. 1). In addition, 184 or 1374 bp DNA length polymorphisms in genomic region no. 2, located upstream of the GTP cyclohydrolase I gene, with the Locus_tag = "ABTJ_01152", associated in 94% with susceptibility to imipenem, was identified. Finally, the highest genetic diversity, determined within the DNA polymerase III subunit gamma/tau gene, can be recommended for future genotyping of multidrug-resistant *A. baumannii* strains. We suggest that the optimized methods, proposed in this report and based on combination of Repeated Sequences and Whole Genome Alignment Differential Analysis (RS&WGADA), can be useful in epidemiological studies concerning specific strains of pathogenic bacteria present in investigated hospitals.

## Data Availability

Not applicable.
